# Widely Targeted Metabolomics Reveals Metabolic Differences Between Yellow and Brown Flaxseed Cultivars (*Linum usitatissimum* L.)

**DOI:** 10.3390/foods15152690

**Published:** 2026-07-30

**Authors:** Wenjuan Li, Jing Sun, Yang Yang, Wei Zhao, Zhao Dang, Yaping Xie, Yanni Qi, Xingrong Wang, Yanjun Zhang, Gang Wang, Nan Lu, Yaming Niu, Zuyu Hu, Limin Wang, Jianping Zhang

**Affiliations:** 1 Institute of Crop Research, Gansu Academy of Agricultural Sciences, Lanzhou 730070, China; liwenjuan@gsagr.ac.cn (W.L.);; 2 Institute of Food Science and Technology, Chinese Academy of Agricultural Sciences, Key Laboratory of Agro-Products Processing, Ministry of Agriculture and Rural Affairs, Beijing 100193, China; 3 Institute of Western Agriculture, Chinese Academy of Agricultural Sciences, Changji 831100, China; 4 Key Laboratory of Crop Gene Resources and Germplasm Innovation in Northwest Cold and Arid Regions, Ministry of Agriculture and Rural Affairs, Lanzhou 730070, China

**Keywords:** flaxseed, metabolomics, polyphenols, seed coat color

## Abstract

Flaxseed (*Linum usitatissimum* L.) is widely recognized as a functional food due to its rich nutritional composition; however, the metabolic basis underlying seed coat color variation remains unclear. In this study, we performed a broad-targeted metabolomics analysis of six flaxseed varieties (three brown and three yellow) grown at two distinct geographical locations. A total of 1456 metabolites were identified, among which flavonoids and phenolic acids were the most abundant differentially accumulated compounds. Eight metabolites consistently exhibited the largest differences across all comparison groups, and thus warrant further investigation as candidate markers in genetically controlled populations. Correlation analysis further indicated that these metabolites are associated with seed coat pigmentation and other developmental processes. Pathway enrichment revealed that the biosynthesis of caffeic acid derivatives, ferulic acid derivatives, and flavones/flavonols was differentially regulated between yellow and brown seeds. Collectively, our findings provide a comprehensive metabolomic landscape of colored flaxseeds and provide a foundation for future studies aimed at breeding flaxseed varieties with targeted color and nutritional traits.

## 1. Introduction

Flax (*Linum usitatissimum* L.) has been cultivated for over two thousand years. Its Latin name means “very useful” [1]. Its seeds are recognized as a functional food because of their exceptionally high content of α-linolenic acid (ALA), short-chain polyunsaturated fatty acids (SC-PUFAs), the lignan secoisolariciresinol diglycoside (SDG), and dietary fiber [2,3,4,5,6]. Additionally, flaxseed contains a variety of antioxidant and anti-cancer bioactive ingredients and has been termed a “superfood” [7]. These excellent nutritional properties have made flaxseed a popular dietary ingredient. Whole grains, granules, or powders prepared from flaxseed can be added to foods, and flaxseed can also be processed into functional foods [8] or nutritional supplements such as oil, capsules, and SDG-based products [1].

Accumulating evidence indicates that several flaxseed components are beneficial for human health, particularly in alleviating cardiovascular diseases [9,10], cancer [11,12], diabetes [13,14,15], inflammatory reaction [16], digestive system disorders and obesity [17,18,19,20], perimenopausal symptoms [21], and osteoporosis. Consequently, flaxseed has attracted growing attention from medical researchers and nutritionists, and its health benefits are closely linked to its bioactive ingredients.

Phenolic compounds are natural or synthetic organic substances that significantly affect the oxidative stability, flavor, and color of foods [22]. Their molecular structures contain at least one aromatic ring bearing hydroxyl groups [23]. Natural polyphenols are products of plant secondary metabolism and possess strong antioxidant and anti-inflammatory properties [24,25]. They not only strengthen immunity [26,27] but also reduce the incidence and delay the progression of cardiovascular diseases [28], neurodegenerative diseases [23,24,25,26,27,28,29], and cancers [30]. Fruits, vegetables, beans, nuts, seeds, whole grains, tea, and coffee are the dominating dietary sources of polyphenols [27]. The types and contents of polyphenols vary greatly among plant species and are influenced by factors such as variety, maturity, and growing conditions. Polyphenolic compounds mainly include flavonoids, phenolic acids, lignans, stilbenes, and tannins. Lignans, flavonoids, and phenolic acids are the primary phenolic compounds in flaxseed [16]. Numerous studies have demonstrated the anti-cancer properties of flaxseed in various cancers, including breast, cervical, ovarian, prostate, colon, leukemia, and melanoma cells [31,32], which are largely attributed to its rich ALA and polyphenol content [5,6,7,8,9,10,11,12,13,14,15,16].

Metabolomics is a comprehensive approach for characterizing small-molecule metabolites in biological systems, including their identification and quantification [33]. Recently, LC-MS/MS-based metabolomics has been widely applied for nutritional quality analysis and crop characterization due to its high sensitivity, specificity, and throughput [34,35]. For instance, it has been used to reveal the flavor components of Alpinia oxyphylla fruit and their relationship with bioactive compounds, to uncover the accumulation profiles of phenolic acids and flavonoids in differently colored quinoa grains [36], and to evaluate the nutritional value of rice grains of different colors (white, red, black, and glutinous) [37].

The types and contents of nutrients and bioactive compounds in flaxseed are strongly influenced by genotype and environment. Flaxseed occurs in two main colors: brown and yellow (or golden yellow). The color and biological activity of fruits largely depend on their chemical composition. For example, quinoa grains are rich in phenolic acids and flavonoids, and their contents are strongly influenced by color genotypes. Grain color formation in wheat is determined by the types and contents of flavonoids and anthocyanins [38]. Existing research on bioactive compounds in flaxseed has been mostly limited to specific cultivars and has mainly focused on linolenic acid and lignans. The nutrient content differs between colored flaxseeds [39]. Brown flaxseeds are rich in proteins, fibers, and antioxidant components (especially tocopherols, phenolic substances, and lignans), whereas yellow or golden yellow flaxseeds are rich in PUFAs and mucilage. However, research on the chemical composition of differently colored flaxseeds remains limited, limiting a comprehensive understanding of their nutritional value and chemical basis. Therefore, a detailed analysis of the phytochemical characteristics of differently colored flaxseeds is necessary to better understand how seed coat color correlates with their biological activities.

In this study, we used UPLC-MS/MS-based metabolomics to comprehensively investigate metabolic variations in differently colored flaxseeds, analyze and compare metabolite composition and contents, and compare seed color with metabolite profiles, thereby providing valuable information for the selection of specialized flaxseed varieties. We acknowledge that the six cultivars differ in their overall genetic backgrounds, and our comparisons are accordingly interpreted as differences between yellow and brown cultivar groups rather than as definitive color-specific associations.

## 2. Materials and Methods

### 2.1. Research Materials

**Figure 1:**
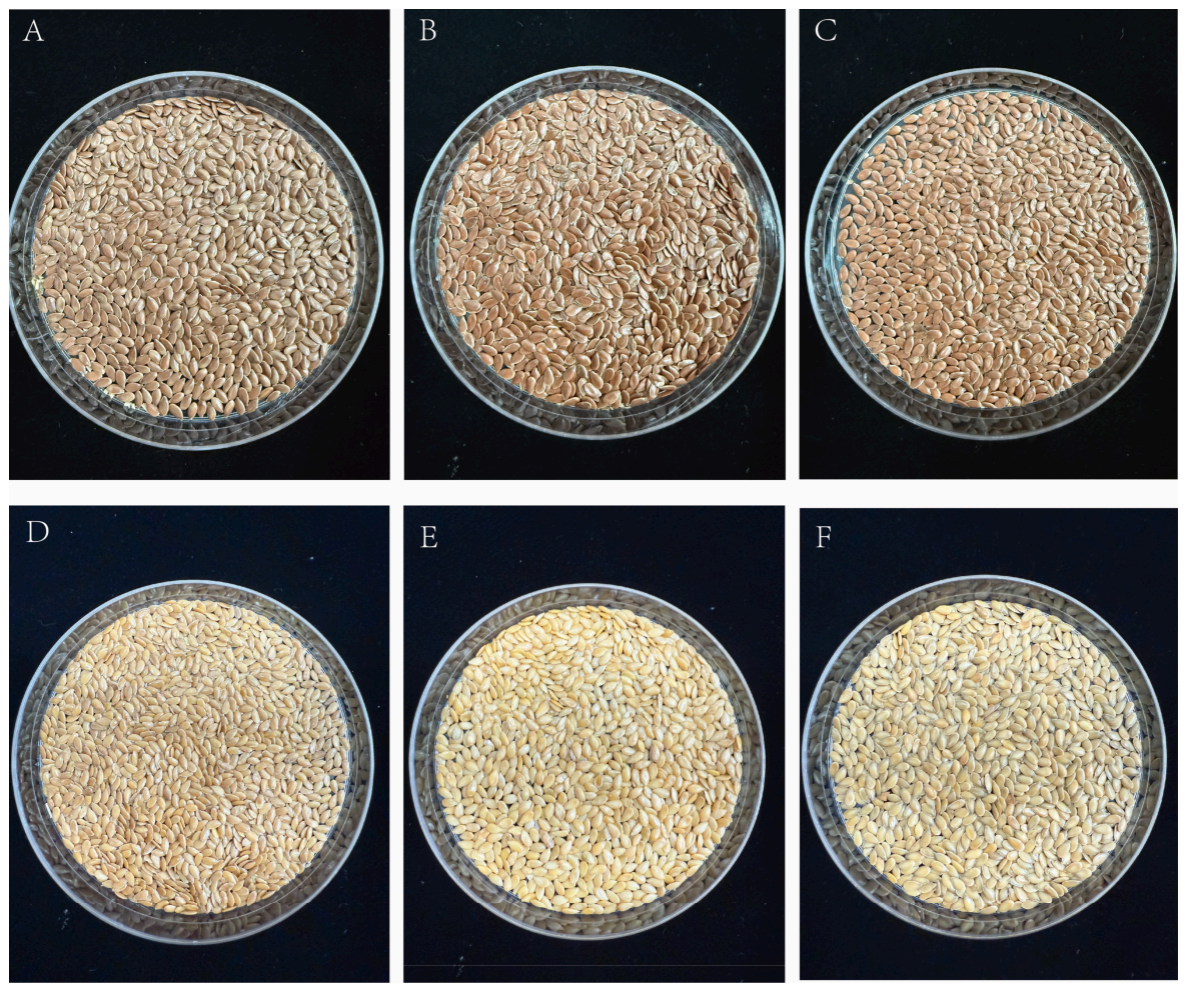
Six flaxseed cultivars with different seed coat colors. Brown flaxseeds: (**A**) 227, (**B**) 141, (**C**) 175; Yellow flaxseeds: (**D**) YJ2435, (**E**) YJ2414, (**F**) YJ2408.

Based on seed coat color, six flax lines were selected from existing germplasm resources, including introduced resources, commercially promoted varieties, and self-bred lines: “Russian-3” (brown, 227), “Longya 15” (brown, 141), “Shuangya 15” (brown, 175), “Baya 21” (yellow, YJ2435), “zh554” (yellow, YJ2414), and “zh232” (yellow, YJ2408) (Figure 1). All lines were grown in Lanzhou New Area (QC, 103°57′ E, 36°16′ N) and Baiyin Jingtai (JT, 104°07′ E, 37°18′ N). Mature flaxseeds were harvested by the Crop Research Institute of Gansu Academy of Agricultural Sciences in 2024 and air-dried at room temperature. All color observations and subsequent metabolomics analyses were performed on intact whole seeds, as the seed coat is the primary tissue responsible for the visible color difference and is not easily separable from the endosperm without affecting metabolite profiles. Three biological replicates and three technical replicates were used for data acquisition and processing. Field experiments were conducted under natural growing conditions at two locations without artificial environmental control. Standard agronomic practices, including irrigation, fertilization, and pest management, were applied uniformly across all plots at each location. Meteorological data were recorded during the growing season; however, comprehensive instrumental records are not available for public access. Based on long-term climatic records for the two regions, Jingtai is characterized by lower annual mean temperature (approximately 8.5 °C vs. 10.5 °C in Lanzhou) and lower annual precipitation (approximately 185 mm vs. 320 mm in Lanzhou), indicating that the two locations represent contrasting environments. While environmental conditions between the two locations differed (e.g., Jingtai is generally drier and cooler than Lanzhou New Area), these differences were not controlled but rather exploited to assess the stability of color-associated metabolites across contrasting environments. The consistent identification of the same color-associated metabolites across these two ecologically distinct sites supports the robustness of our findings.

### 2.2. Reagents and Chemicals

LC-MS grade acetonitrile and formic acid were purchased from Merck (Darmstadt, Germany). Methanol was obtained from Aladdin (Shanghai, China). Other chemical reagents were supplied by Sinopharm Chemical Reagent Co., Ltd. (Shanghai, China).

### 2.3. Sample Preparation and Extraction

Impurities and damaged seeds were removed, and the remaining seeds were dried. The samples were freeze‑dried (Scientz‑100F, Scientz Biotechnology Co., Ltd., Ningbo, China) for 63 h. After grinding (MM 400 grinder, Retsch GmbH, Haan, Germany; 30 Hz, 1.5 min), 50 mg of sample powder was mixed with 1200 μL of pre‑cooled 70% methanol‑water containing an internal standard (1 mg of standard solution dissolved in 1 mL of 70% methanol, then diluted to 250 μg/mL with 70% methanol). The mixture was vortexed six times (30 s each, once every 30 min). After centrifugation (Eppendorf 5810 R, Eppendorf SE, Hamburg, Germany), the supernatant was collected, filtered through a 0.22 μm microporous membrane (Millipore, Burlington, MA, USA), and stored in a sample vial for UPLC‑MS/MS analysis. 

### 2.4. UPLC Conditions

The sample extracts were analyzed by UPLC (ExionLC™ AD, Sciex, Framingham, MA, USA) coupled with a triple quadrupole mass spectrometer (QTRAP® 6500+, Sciex, Framingham, MA, USA). The analysis used an Agilent SB-C18 chromatographic column (1.8 µm, 2.1 mm × 100 mm) and a mobile phase consisting of solvent A (0.1% formic acid in ultrapure water) and solvent B (acetonitrile added with 0.1% formic acid). The sample test was carried out using a gradient program, with the initial parameters setting as: 0.00 min, B 5%, within 9.00 min, B raised to 95% and maintained at 95% for 1 min, 10.00–11.10 min, B declined to 5%, and equilibrated at 5% till 14 min; a flow rate of 0.35 mL/min; column temperature of 40 °C; loading volume of 2 μL.

### 2.5. ESI-Q TRAP-MS Parameters

Electrospray ionization (ESI) temperature was 500 °C; ion spray voltage was 5500 V (positive mode) and −4500 V (negative mode); ion source gas I (GSI), gas II (GSII), and curtain gas (CUR) were 50, 60, and 25 psi, respectively; collision-induced ionization parameters were set to high. QQQ scanning was performed in multiple reaction monitoring (MRM) mode using nitrogen as the collision gas. The optimal declustering potential (DP) and collision energy (CE) for each MRM ion pair were determined. A specific MRM ion pair was monitored at each phase according to the eluted metabolites.

### 2.6. Metabolite Identification and Quantification

Metabolites were identified using a self-built database (MWDB, Metware Biotechnology Co., Wuhan, China) and publicly available metabolite databases. To ensure accurate annotation, isotope signals and adduct signals (K^+^, Na^+^, NH_4_^+^, etc.) were removed during analysis. Metabolite quantification was performed in MRM mode using a triple quadrupole mass spectrometer. The quadrupole rods filtered precursor ions, eliminating interference from non-target ions. Precursor ions were fragmented by collision-induced ionization, and the resulting fragment ions were further filtered to obtain characteristic ions, enabling accurate and reproducible quantification. Chromatographic peak areas were integrated for all metabolites, and peak area corrections were applied across different samples.

### 2.7. Statistical Analysis

MS data were processed using Analyst 1.6.3 software (AB Sciex). Metabolite qualitative and quantitative analyses were performed using the MWDB database. Chromatographic peaks were calibrated by retention time and peak shape to ensure accurate qualitative and quantitative comparisons. Filtered and processed data were subjected to principal component analysis (PCA), hierarchical clustering analysis (HCA), and orthogonal partial least squares discriminant analysis (OPLS-DA) using R scripts. Differential metabolites among groups were initially selected based on variable importance in projection (VIP) values from the OPLS-DA model (biological replicates ≥ 3). Differential metabolites were screened using criteria of VIP ≥ 1, |log_2_(FC)| ≥ 1, and adjusted *p*-value (FDR) < 0.05. The false discovery rate (FDR) was controlled using the Benjamini–Hochberg procedure to adjust for multiple comparisons. Venn diagrams were used to visualize relationships among differential metabolites across groups. KEGG pathway enrichment analysis was performed to identify pathways containing differential metabolites. All results are presented as mean ± SD of three replicates. Graphs were drawn using GraphPad Prism 8 and the Metware Cloud platform (https://cloud.metware.cn).

To statistically validate the differential accumulation of candidate metabolites, a two-way mixed-effects ANOVA was performed using R (lmer function in the lme4 package), with seed coat color (brown vs. yellow) and growing location (Lanzhou vs. Jingtai) as fixed factors, their interaction as a fixed effect, and genotype nested within color as a random factor. Metabolite abundance data were log_2_-transformed prior to analysis to meet the assumptions of normality (Shapiro–Wilk test) and homogeneity of variance (Levene’s test). For metabolites showing significant color effects, Tukey’s HSD post hoc test was conducted for mean comparisons (α = 0.05). *p*-values from multiple comparisons were adjusted using the Benjamini–Hochberg false discovery rate (FDR) procedure (q < 0.05).

## 3. Results and Discussion

### 3.1. Overview of the Metabolome of Differently Colored Flaxseeds

Based on seed color differences, three brown (227, 141, 175) and three yellow (YJ2435, YJ2408, YJ2414) flaxseed varieties were selected as materials (Figure 1). These varieties were grown in two locations: Lanzhou New Area (QC) and Baiyin Jingtai (JT). Metabolites from the twelve flaxseed samples were analyzed using the UPLC-ESI-MS/MS platform. The total ion chromatogram (TIC) and extracted ion chromatogram (XIC) of quality control (QC) samples in MRM mode are shown in Supplementary Figure S1. TIC was used to evaluate the consistency of metabolite extraction and detection, while the multi-peak XIC map displayed detectable substances in the samples; each chromatographic peak of a different color represents a detectable metabolite. High correlation coefficients were observed among the three biological replicates of each cultivar in Lanzhou (R^2^ = 0.965–0.995) and Jingtai (R^2^ = 0.890–0.994) (Figure S2), indicating good sample homogeneity.

**Figure 2:**
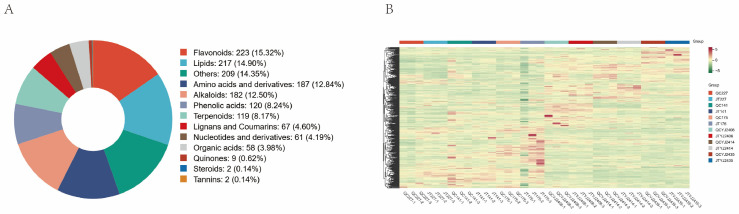
Overall evaluation based on all detected metabolites: (**A**) Classification of the 1456 metabolites detected in differently colored flaxseeds; (**B**) Hierarchical clustering heatmap of all metabolites. Each column represents a sample, and each row represents a metabolite. Red indicates relatively high abundance, green indicates low abundance.

The types and proportions of metabolites differed among cultivars. Notably, A total of 1456 metabolites were detected, including 223 flavonoids (15.32%), 217 lipids (14.90%), 187 amino acids and derivatives (12.84%), 182 alkaloids (12.50%), 120 phenolic acids (8.24%), 119 terpenoids (8.17%), 61 nucleotides and derivatives (4.20%), 58 organic acids (3.98%), 45 lignans (3.09%), 22 coumarins (1.51%), 9 quinones (0.62%), 2 tannins (0.14%), 2 steroids (0.14%), and 209 other compounds (14.35%) (Figure 2A). Notably, the proportion of polyphenolic compounds was relatively high (28.3%), with flavonoids, phenolic acids, lignans, coumarins, and tannins accounting for a considerable share of all metabolites. In QC227, 1329 metabolites were identified, whereas 1348, 1341, 1338, 1313, and 1288 metabolites were detected in QC175, QC141, QCYJ2408, QCYJ2414, and QCYJ2435, respectively. In JT227, 1348 metabolites were detected, while 1355, 1346, 1352, 1307, and 1323 metabolites were screened in JT175, JT141, JTYJ2408, JTYJ2414, and JTYJ2435 (Table S1). These results demonstrate significant differences in the metabolic profiles among the twelve samples.

### 3.2. Multivariate Statistical Analysis

To comprehensively evaluate metabolic differences associated with seed coat color and growing environment, the six flaxseed cultivars were organized into six pairwise comparison groups based on seed color (yellow vs. brown) within each location: QC2435 vs. QC227, QC2414 vs. QC141, QC2408 vs. QC175 for the Lanzhou (QC) site, and JT2435 vs. JT227, JT2414 vs. JT141, JT2408 vs. JT175 for the Jingtai (JT) site. Principal component analysis (PCA), hierarchical clustering analysis (HCA), and orthogonal partial least squares discriminant analysis (OPLS-DA) were employed to assess inter-and intra-group variability.

PCA of QC (Lanzhou New Area) samples showed that PC1 and PC2 explained 24.48% and 14.27% of the total variance, respectively (cumulative 38.75%) (Figure S3A). For the JT samples, PC1 and PC2 accounted for 24.1% and 15.05% of the variance, respectively, with a cumulative contribution of 39.15% (Figure S3B). In both locations, the six cultivars formed clearly separated clusters, and the three biological replicates of each cultivar clustered tightly, indicating high reproducibility and significant metabolic differentiation among the six flaxseed lines. These results confirm the suitability of the dataset for subsequent qualitative and quantitative analyses.

The HCA heatmap (Figure 2B) further illustrated the metabolic relationships among samples. The six cultivars were distinctly grouped, and a clear separation between brown and yellow seeds was observed, suggesting that seed coat color is closely associated with metabolic composition. Notably, cultivars of the same color but from different environments also clustered together, indicating that genetic factors play a dominant role in shaping the metabolite profiles, with environmental influences being secondary.

**Figure 3:**
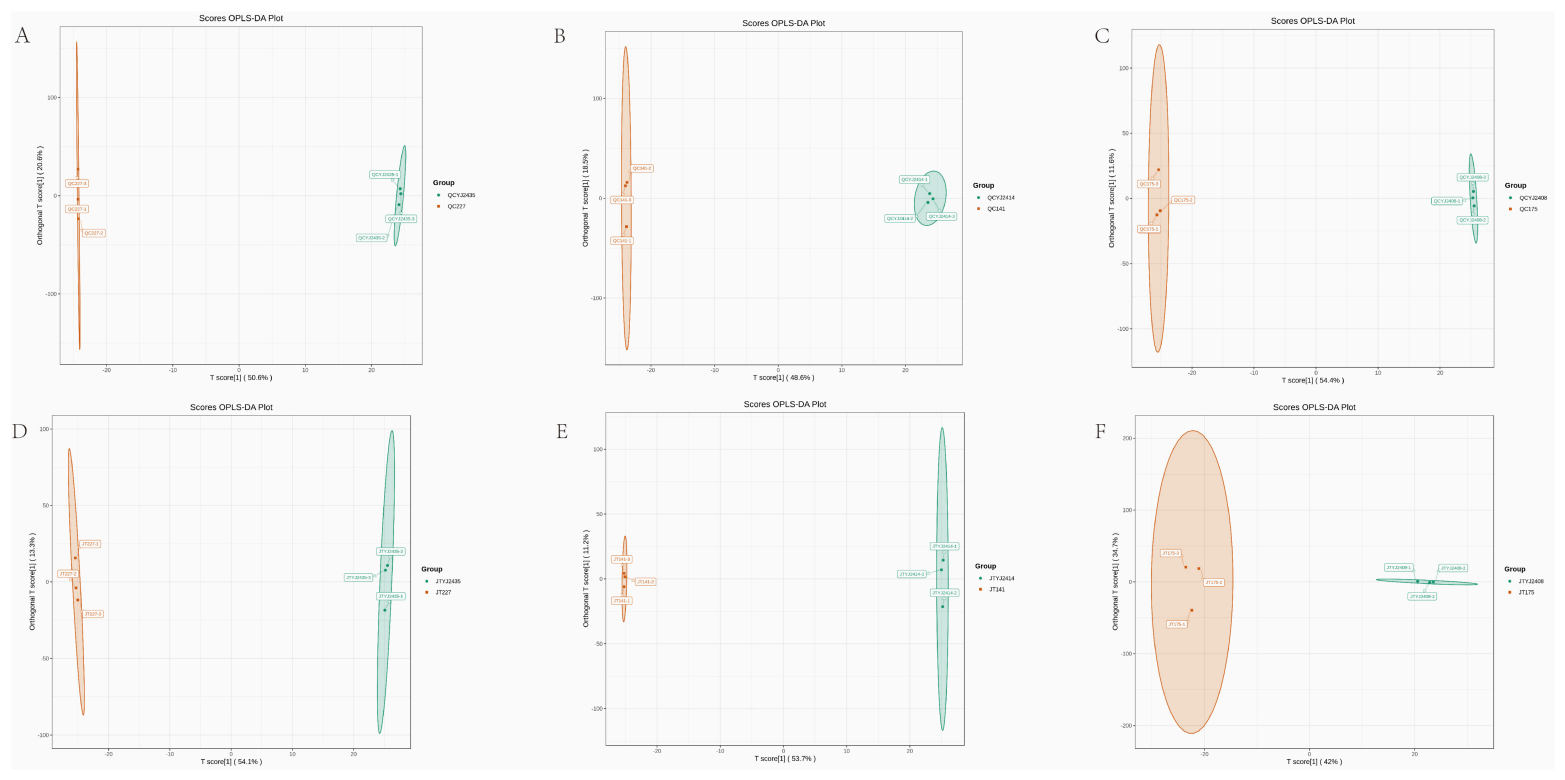
OPLS-DA score plots for pairwise comparisons. (**A**) QC2435 vs. QC227, (**B**) QC2414 vs. QC141, (**C**) QC2408 vs. QC175, (**D**) JT2435 vs. JT227, (**E**) JT2414 vs. JT141, (**F**) JT2408 vs. JT175.

OPLS-DA models were constructed to maximize class separation and to identify metabolites contributing to discrimination. The quality parameters for each model were as follows: QC2435 vs. QC227 (R^2^X = 0.712, R^2^Y = 1, Q^2^ = 0.956); QC2414 vs. QC141 (R^2^X = 0.671, R^2^Y = 1, Q^2^ = 0.943); QC2408 vs. QC175 (R^2^X = 0.660, R^2^Y = 1, Q^2^ = 0.965); JT2435 vs. JT227 (R^2^X = 0.675, R^2^Y = 1, Q^2^ = 0.953); JT2414 vs. JT141 (R^2^X = 0.648, R^2^Y = 1, Q^2^ = 0.960); and JT2408 vs. JT175 (R^2^X = 0.767, R^2^Y = 0.997, Q^2^ = 0.936). All models exhibited R^2^Y and Q^2^ values exceeding 0.9, indicating excellent stability and predictive ability (Figure S4). The score plots of OPLS-DA (Figure 3A–F) displayed clear separation between the two color groups in each pairwise comparison, with no overlap between brown and yellow seeds. This distinct separation underscores that the metabolic profiles are strongly influenced by seed coat color, while environmental factors (location) do not obscure the color-associated differences. The high Q^2^ values also confirm that the models are not overfitted, making them reliable for subsequent differential metabolite screening.

### 3.3. Differential Metabolite Analysis

**Figure 4:**
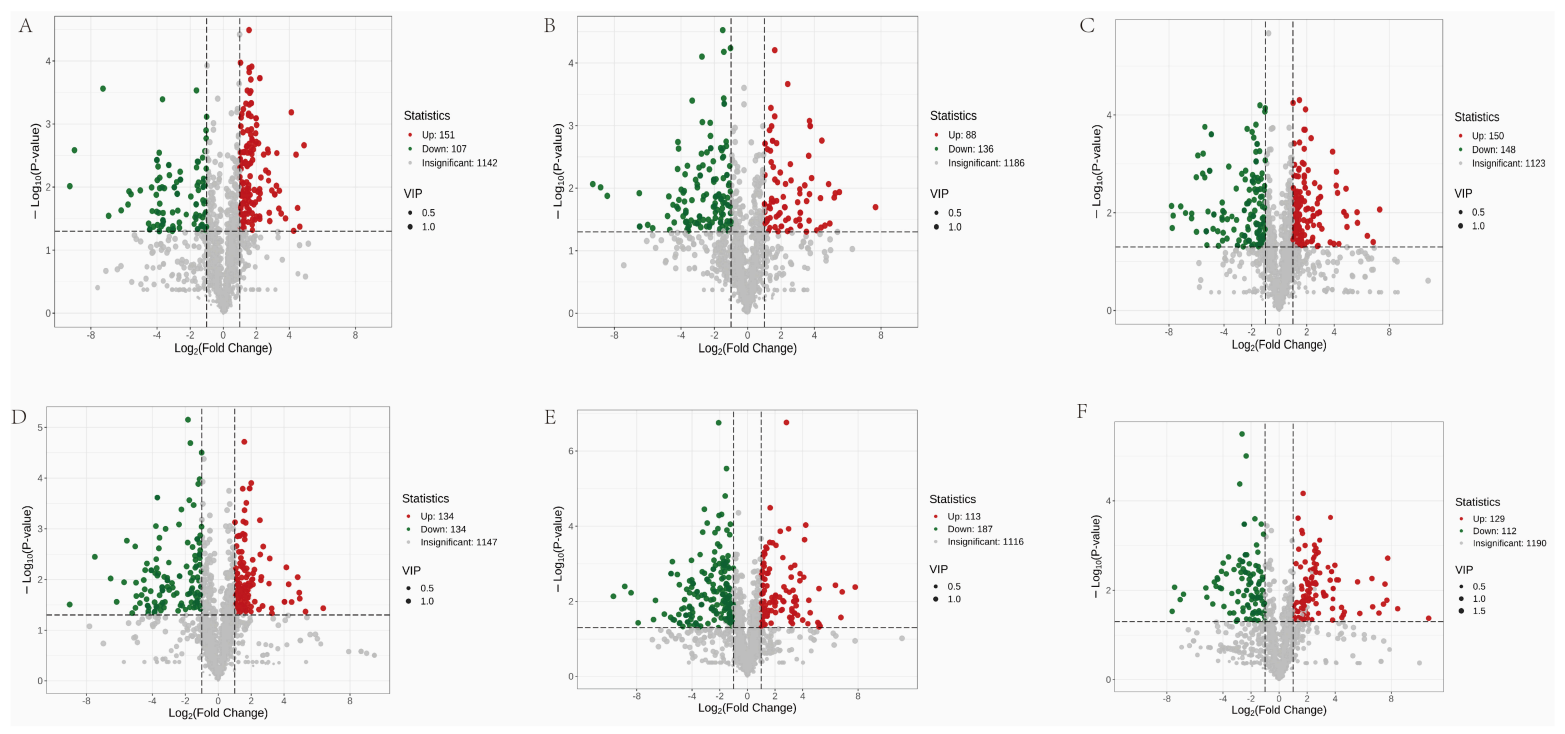
(**A**–**F**) Volcano plots show the differences in metabolite levels among the six groups. Red, blue, and gray dots refer to over-expressed, under-expressed, and insignificant differentially expressed metabolites, correspondingly. (**A**) QC YJ2435 vs. QC227; (**B**) QC YJ2414 vs. QC141; (**C**) QC YJ2408 vs. QC175; (**D**) JT YJ2435 vs. JT227; (**E**) JT YJ2414 vs. JT141; (**F**) JT YJ2408 vs. JT175.

Differential metabolites were screened using criteria of VIP ≥ 1, |log_2_(FC)| ≥ 1, and FDR-adjusted *p*-value < 0.05 (Benjamini–Hochberg). Volcano plots summarizing the results are shown in Figure 4A–F. A total of 376 differential metabolites (202 up-regulated, 174 down-regulated) were identified between QC YJ2435 and QC 227 (Figure 4A, Table S2); 401 (144 up, 257 down) between QC YJ2414 and QC 141 (Figure 4B); and 429 (229 up, 200 down) between QC YJ2408 and QC 175 (Figure 4C). For the JT groups, 356 differential metabolites (170 up, 186 down) were found between JT YJ2435 and JT 227 (Figure 4D); 417 (152 up, 265 down) between JT YJ2414 and JT 141 (Figure 4E); and 390 (185 up, 205 down) between JT YJ2408 and JT 175 (Figure 4F).

The types and numbers of differential metabolites varied among cultivars. The differential metabolites in the six comparison groups were classified into 10–11 categories (Figure S5A–F), mainly including phenolic acids, flavonoids, lignans, coumarins, alkaloids, organic acids, amino acids and derivatives, lipids, and terpenoids. Notably, the most significantly differentially expressed metabolites were polyphenolic compounds, especially flavonoids and phenolic acids. Therefore, we focused on the polyphenolic profiles of different flaxseed cultivars.

Comparative analysis of QC YJ2435 vs. QC227 showed that the fold changes of 153 polyphenolic compounds ranged from 0.002 to 35.38 (Table S3). In QC YJ2414 vs. QC141, 177 polyphenolic compounds were detected with FC values ranging from 0.002 to 203.61. Between QC YJ2408 and QC175, 171 polyphenolic compounds had FC values from 0.004 to 61.51. Between JT YJ2435 and JT227, 153 polyphenolic compounds had FC values from 0.002 to 514.83; between JT YJ2414 and JT141, 214 polyphenolic compounds had FC values from 0.001 to 115.82; and between JT YJ2408 and JT175, 159 polyphenolic compounds had FC values from 0.005 to 1032.57.

Our results indicate a significant difference in polyphenolic compounds between yellow and brown flaxseeds, suggesting that polyphenolic content may be strongly associated with flaxseed color. We screened specific metabolites in differently colored flaxseeds by analyzing metabolomic changes among cultivars.

### 3.4. Key Differential Metabolites Between Yellow and Brown Cultivar Groups

**Figure 5:**
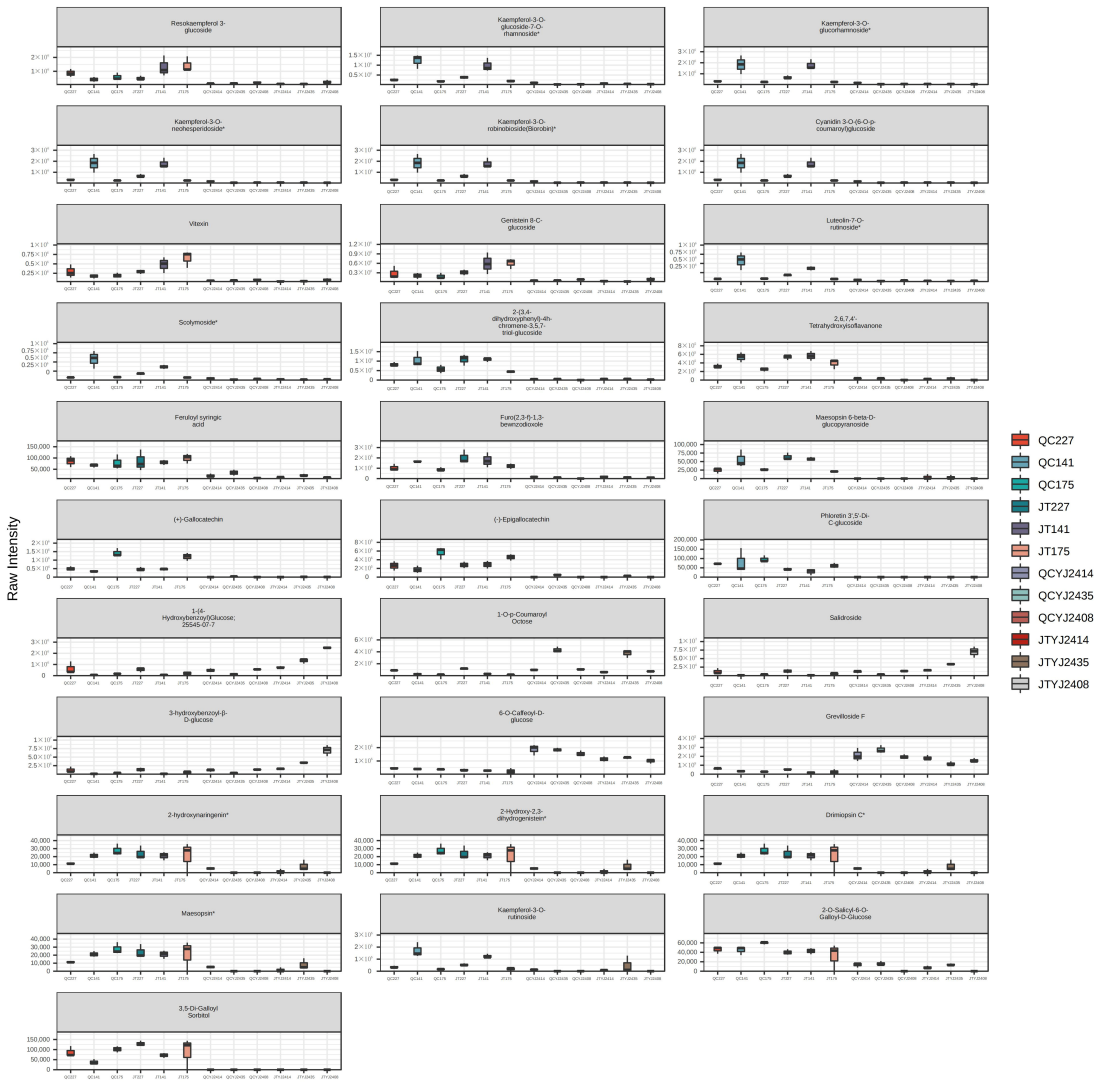
Relative contents of 31 important phenolic metabolites involved in flaxseed color differentiation. Duncan’s test was used to assess significance. Different letters indicate significant differences (*p* < 0.05) based on three replicate values. Asterisk (*) indicate metabolites that were consistently detected at significantly higher levels in brown seeds compared to yellow seeds across all comparison groups. The Y‑axis shows the relative content (peak area) of each compound.

We analyzed the polyphenolic compounds across the six comparison groups and identified 31 common differential metabolites (Figure S6), including 21 flavonoids and 10 phenolic acids (Table 1). Among them, two flavonoids (Maesopsin 6-β-D-glucopyranoside and Phloretin 3′,5′-di-C-glucoside) and one phenolic acid (3,5-di-galloyl sorbitol) were detected only in brown seeds and were all up-regulated in brown seeds (Figure 5, Table S4). The concentration of Maesopsin 6-β-D-glucopyranoside in brown seeds was 10.68- to 36.40-fold higher than in yellow seeds; Phloretin 3′,5′-di-C-glucoside was 63.74- to 207.74-fold higher; and 3,5-di-galloyl sorbitol was 52.08- to 180.09-fold higher.

In addition, four compounds showed significantly higher concentrations in brown seeds than in yellow seeds: the flavanols (−)-epigallocatechin, leucocyanidin, (+)-gallocatechin, and the phenolic compound gallate (Figure 5, Table S4). The concentrations of (−)-epigallocatechin, leucocyanidin, (+)-gallocatechin, and gallate in brown seeds were 4.97- to 217.17-fold, 14.31- to 518.81-fold, 12.45- to 823.46-fold, and 42.24- to 510.98-fold higher than in yellow seeds, respectively.

In addition to anthocyanins, flavonoids are well-known compounds associated with plant coloration. Numerous studies have shown that the biosynthesis of phenylpropanoids, flavonoids, isoflavones, flavonols, and anthocyanins is highly relevant to plant color formation [35,36,37,38,39,40,41]. Although phenolic acids are not directly used as chromogenic pigments, they can markedly affect the color of organs such as flowers and seeds through various indirect mechanisms. Phenolic acids can enhance color by 19–27%, and gallate shows the strongest protective effect on color [42]. Existing studies have indicated key co-pigmentation of anthocyanins with co-pigments, flavonoids and phenolic acid compounds [43,44], a mechanism that stabilizes color by protecting anthocyanin pigments from nucleophilic attack. Therefore, we hypothesize that the seven flavonoids and phenolic acid compounds described above act as important co-pigments and may synergistically contribute to the brown color of flaxseed.

Using brown flaxseed as the control, among the top 10 compounds most up-regulated in yellow seeds, one lignan compound, demethoxypinoresinol, showed a markedly higher concentration in yellow seeds (13.87- to 28.95-fold) than in brown seeds (Table S4). We propose that this lignan may be associated with the yellow color of flaxseed, though this hypothesis requires further experimental verification.

### 3.5. KEGG Analyses on Differential Metabolites

**Figure 6:**
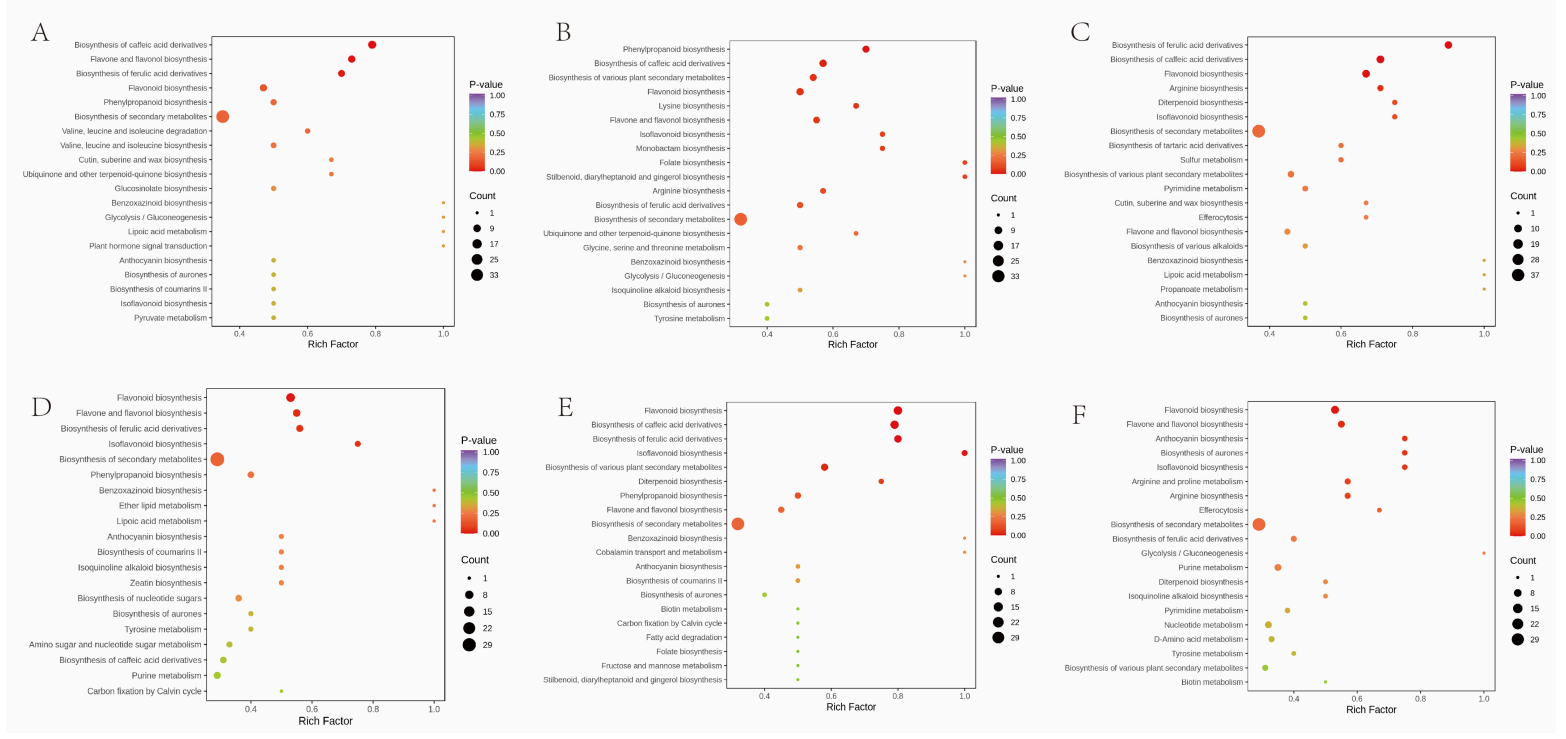
KEGG pathway enrichment bubble plots. (**A**) QC YJ2435 vs. QC227, (**B**) QC YJ2414 vs. QC141, (**C**) QC YJ2408 vs. QC175, (**D**) JT YJ2435 vs. JT227, (**E**) JT YJ2414 vs. JT141, (**F**) JT YJ2408 vs. JT175. Each bubble represents a metabolic pathway; the horizontal axis indicates the impact factor (bubble size). The bubble color represents the *p*-value of enrichment (lighter color indicates a lower enrichment level).

The KEGG database was used to investigate signaling pathways and metabolite accumulation. Differential metabolites from all comparison groups were annotated and enriched. The number of enriched KEGG pathways for each comparison was: QC YJ2435 vs. QC227 (61 pathways), QC YJ2414 vs. QC141 (66), QC YJ2408 vs. QC175 (71), JT YJ2435 vs. JT227 (65), JT YJ2414 vs. JT141 (65), and JT YJ2408 vs. JT175 (63). The dominant pathways are shown in bubble plots (Figure 6).

Significantly enriched pathways in QC YJ2435 vs. QC227 were biosynthesis of caffeic acid derivatives, flavone and flavonol biosynthesis, and biosynthesis of ferulic acid derivatives (*p* < 0.05). In QC YJ2414 vs. QC141, significantly enriched pathways included phenylpropanoid biosynthesis, biosynthesis of caffeic acid derivatives, and biosynthesis of various plant secondary metabolites (*p* < 0.05). In QC YJ2408 vs. QC175, the pathways were biosynthesis of ferulic acid derivatives, biosynthesis of caffeic acid derivatives, flavonoid biosynthesis, and arginine biosynthesis (*p* < 0.05). In JT YJ2435 vs. JT227, flavonoid biosynthesis, flavone and flavonol biosynthesis, and biosynthesis of ferulic acid derivatives were enriched (*p* < 0.05). In JT YJ2414 vs. JT141, flavonoid biosynthesis, biosynthesis of caffeic acid derivatives, biosynthesis of ferulic acid derivatives, isoflavonoid biosynthesis, and biosynthesis of diverse plant secondary metabolites were enriched (*p* < 0.05). In JT YJ2408 vs. JT175, flavonoid biosynthesis and flavone/flavonol biosynthesis were enriched (*p* < 0.05). These results suggest that the biosynthesis pathways of caffeic acid derivatives, flavonoids, flavones/flavonols, and ferulic acid derivatives may be associated with the color and nutritional components of flaxseeds, consistent with the finding that most differential metabolites were phenolic acids and flavonoids.

The consistent enrichment of flavonoid biosynthesis and flavone/flavonol biosynthesis pathways across all six comparison groups highlights the central role of this metabolic route in differentiating yellow and brown flaxseeds. Flavonoids are synthesized via the phenylpropanoid pathway, with chalcone synthase (CHS) catalyzing the first committed step, followed by a series of enzymatic reactions involving chalcone isomerase (CHI), flavanone 3-hydroxylase (F3H), and flavonol synthase (FLS). The differential accumulation of specific flavanols (e.g., (−)-epigallocatechin and leucocyanidin) in brown seeds suggests that the flux through the flavonoid pathway may preferentially accumulate toward condensed tannin precursors in brown genotypes. Conversely, the relative enrichment of demethoxypinoresinol in yellow seeds may indicate a metabolic shift toward lignan biosynthesis, potentially competing with flavonoid production for common precursors. These observations warrant further investigation at the transcriptional and enzymatic levels to identify the key regulatory nodes controlling the partitioning of carbon flux between flavonoid and lignan pathways.

### 3.6. Correlation Analyses Between Differential Metabolites and Quality Traits

**Figure 7:**
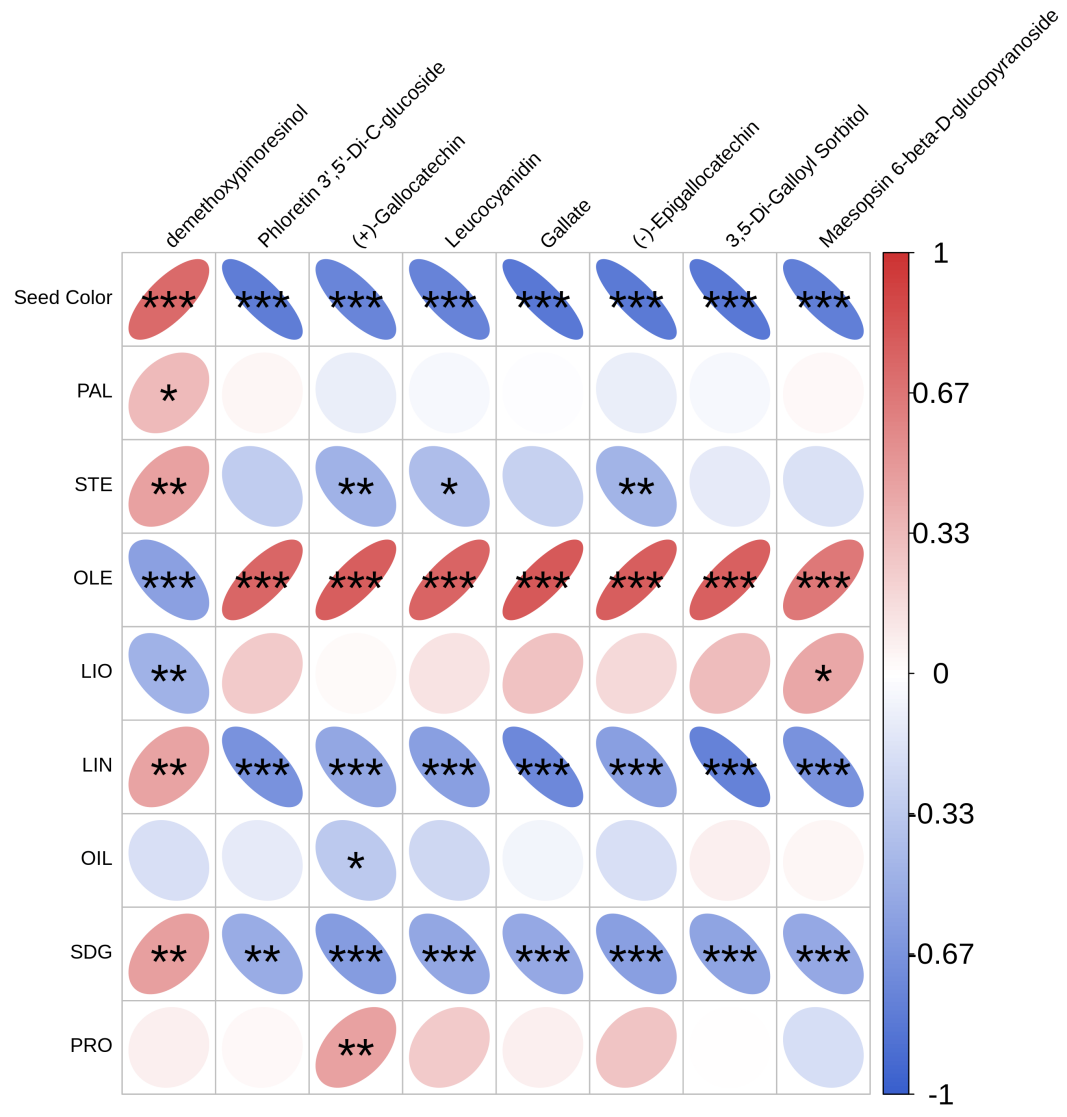
Correlation analyses between differential metabolites and quality traits. The abscissa represents the differential metabolites. * represents significant difference (* 0.01 < *p* < 0.05; ** 0.001 < *p* < 0.01; *** *p* < 0.001).

Small-molecule metabolism closely reflects plant function and phenotype. Therefore, we performed correlation analysis between the contents of eight differential metabolites (selected from differently colored flaxseed cultivars grown in different environments) and associated traits, including seed coat color, fatty acid content (palmitic, stearic, oleic, linoleic, and linolenic acids), oil content, protein content, and lignan content (Figure 7). The lignan demethoxypinoresinol, which was significantly up-regulated in yellow seeds, was highly positively correlated with seed coat color and significantly positively correlated with linolenic acid. The other seven compounds showed strong negative correlations with seed coat color, suggesting that these eight compounds may be associated with seed coat pigmentation. Among them, one flavanol, (+)-gallocatechin, was strongly positively correlated with oleic acid and protein. The other two flavanols, (−)-epigallocatechin and leucocyanidin, also showed strong positive associations with oleic acid and protein. The flavonoids (phloretin 3′,5′-di-C-glucoside and maesopsin 6-β-D-glucopyranoside) and phenolic acids (3,5-di-galloyl sorbitol and gallate) were extremely positively correlated with oleic acid. Accordingly, these eight compounds may also participate in other metabolic processes during seed development. Based on these findings, eight compounds—five flavonoids, two phenolic acids, and one lignan—were identified as closely associated with flaxseed coat color and are candidates for future research.

This comparative analysis of the metabolite profiles of differently colored flaxseed cultivars is of fundamental importance for the quality assessment of flaxseed germplasm. In addition, due to their antioxidant capacity and significant anti-bacterial, anti-viral, anti-atherosclerotic, and anti-hypertensive effects, polyphenolic compounds have received extensive attention from pharmacologists and other experts in recent years. The marked differences in polyphenolic compounds among differently colored flaxseed cultivars also lay the foundation for their diverse applications in functional foods.

Although the two growing locations (Lanzhou and Jingtai) differ in altitude, temperature, and soil conditions, the core set of candidate metabolites identified in this study remained consistent across both environments. This consistency suggests that the metabolic differences between yellow and brown seeds are robust and primarily driven by genetic factors, with environmental variation exerting a secondary modulatory effect. Notably, several metabolites showed location-specific abundance differences, indicating that environmental conditions can fine-tune the accumulation of certain polyphenolic compounds. Future studies with multi-year and multi-location trials would be valuable to further assess the stability of these color-associated metabolic markers. We acknowledge that our study was conducted in a single growing season, and annual environmental variation may influence metabolite accumulation. Therefore, multi-year trials are needed to validate the stability of the identified markers.

We acknowledge that the six flaxseed varieties used in this study differ not only in seed coat color but also in their overall genetic backgrounds. Ideally, a near-isogenic or mutant pair differing only in seed color would allow a cleaner dissection of color-specific metabolic effects. However, we note that (a) the metabolic profiles of the three brown varieties clustered closely together, distinct from the three yellow varieties, despite their diverse genetic origins; (b) the same candidate metabolites were consistently identified across two distinct geographical locations; and (c) hierarchical clustering based on the full metabolome separated samples primarily by color rather than by genotype. These observations suggest that seed coat color, rather than genotype per se, is a major factor contributing to the observed metabolic differences. Nevertheless, future studies using near-isogenic lines or bi-parental populations segregating for seed color are essential to unequivocally validate the color-association of the candidate metabolites identified here.

In addition, we acknowledge that our classification of seed coat color was based on categorical visual assessment rather than quantitative colorimetry (e.g., CIE Lab). Quantitative color phenotyping would enable more precise correlation analyses between color parameters and metabolite abundance, and should be incorporated in future studies. The seed samples from this study were entirely consumed during metabolomics extraction and are no longer available for additional color measurements. Nevertheless, the consistent separation of yellow and brown groups in PCA and HCA (Figure 2B and Figure S3) supports the validity of the categorical classification as a meaningful biological discriminator for the purposes of this exploratory study.

### 3.7. ANOVA Validation of Candidate Metabolites

**Figure 8:**
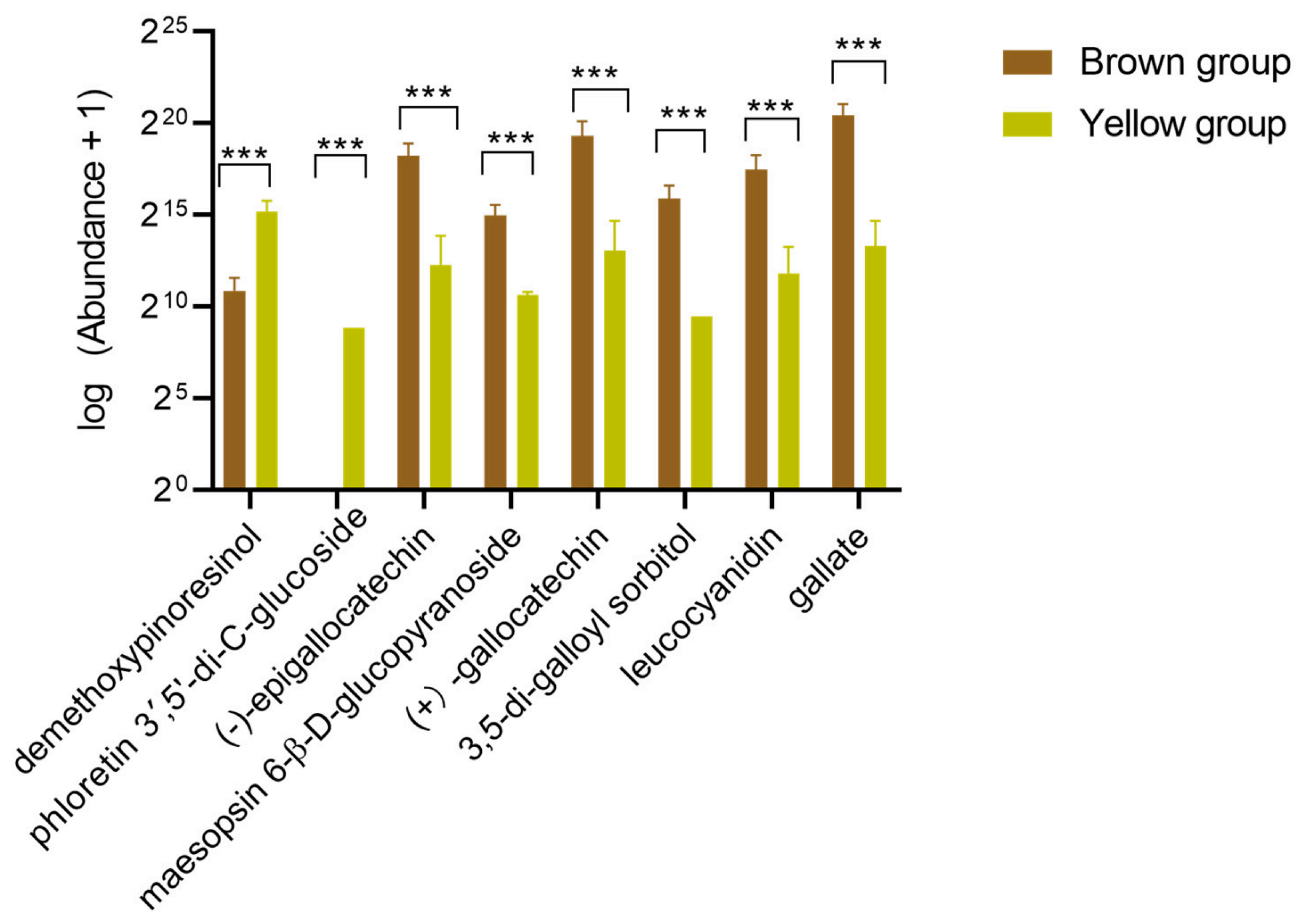
Comparison of the eight candidate metabolite abundances between brown and yellow flaxseed cultivars. Data are presented as mean ± SD (*n* = 6 per color group, combining two locations). Metabolite abundance values were log_2_-transformed prior to plotting. Asterisks indicate significant differences between brown and yellow groups (Tukey HSD: *** *p* < 0.001). Metabolites: demethoxypinoresinol; phloretin 3′,5′-di-C-glucoside; (−)-epigallocatechin; maesopsin 6-β-D-glucopyranoside; (+)-gallocatechin; 3,5-di-galloyl sorbitol; leucocyanidin; gallate.

To validate the differential accumulation of the eight candidate metabolites using standard statistical methods, a two-way mixed-effects ANOVA was performed with seed coat color (brown vs. yellow) and growing location (Lanzhou vs. Jingtai) as fixed factors and genotype nested within color as a random factor. The ANOVA results (Table 2) confirmed that seed coat color had a significant effect on all eight candidate metabolites after FDR correction (q < 0.05). Seven metabolites (maesopsin 6-β-D-glucopyranoside, phloretin 3′,5′-di-C-glucoside, 3,5-di-galloyl sorbitol, (−)-epigallocatechin, (+)-gallocatechin, leucocyanidin, and gallate) were significantly higher in brown seeds than in yellow seeds (Tukey HSD, *p* < 0.05), whereas Demethoxypinoresinol was significantly higher in yellow seeds (Tukey HSD, *p* < 0.05). No significant location effects or color × location interaction effects were detected for most metabolites (*p* > 0.05), indicating that the color-associated differences were consistent across the two growing environments. The bar plot (Figure 8) visually demonstrates these differences, showing that all eight metabolites exhibited clear separation between brown and yellow groups. Together, the ANOVA results and the visual comparison (Table 2; Figure 8) provide statistical confirmation that the eight candidate metabolites robustly differentiate brown and yellow flaxseed cultivars.

## 4. Conclusions

In this study, we performed a comprehensive broad-targeted metabolomics analysis of six flaxseed varieties differing in seed coat color (brown vs. yellow) and grown in two distinct environments. A total of 1456 metabolites were identified, revealing that the metabolome of flaxseed is highly diverse and significantly influenced by seed color. Flavonoids and phenolic acids were the dominant differential metabolite classes, highlighting the pivotal role of the phenylpropanoid pathway in color-related metabolic variation.

Through rigorous multivariate analysis and cross-comparison of six yellow-brown pairs, we identified eight metabolites that consistently showed differences between the yellow and brown cultivar groups. Among these, three compounds—maesopsin 6-β-D-glucopyranoside, phloretin 3′,5′-di-C-glucoside, and 3,5-di-galloyl sorbitol—were exclusively or highly accumulated in brown seeds, warranting further investigation as candidate biomarkers for brown seed coat color in future genetically controlled studies. Four flavanols ((−)-epigallocatechin, leucocyanidin, (+)-gallocatechin) and gallate were also markedly enriched in brown seeds, supporting a synergistic role of flavanols and phenolic acids in brown pigmentation via co-pigmentation mechanisms. Conversely, the lignan demethoxypinoresinol was specifically elevated in yellow seeds, suggesting a previously unrecognized association between lignan metabolism and yellow coloration.

Pathway enrichment analysis consistently highlighted the biosynthesis of caffeic acid derivatives, ferulic acid derivatives, and flavones/flavonols as the most significantly enriched pathways in comparisons. These findings indicate that the differential accumulation of color-associated metabolites is likely driven by coordinated regulation of key enzymatic steps within the phenylpropanoid and flavonoid biosynthetic routes.

Correlation analysis further revealed that most brown-associated phenolic compounds were positively correlated with oleic acid and protein content, implying that selection for brown seed coat color might concurrently enhance nutritional quality traits. Conversely, the yellow-associated lignan demethoxypinoresinol showed a positive correlation with linolenic acid, suggesting a metabolic link between lignan and polyunsaturated fatty acid metabolism.

Taken together, our results provide a comprehensive metabolomic resource for flaxseed color differentiation, identify candidate metabolites for seed coat color and offer new insights into the metabolic networks that connect pigmentation with nutritional quality. These findings provide a useful reference for marker-assisted breeding of flaxseed varieties with desirable color and functional properties, and open new avenues for investigating the molecular mechanisms linking lignan metabolism to pigmentation. While our results reveal strong correlations between specific metabolites and seed coat color, further functional studies (e.g., genetic transformation or in vitro pigment reconstitution) are required to establish direct causative relationships. Nevertheless, the robust and consistent metabolic markers identified in this study provide a useful basis for future marker-assisted breeding of flaxseed varieties with desirable color and functional properties.:

## Figures and Tables

**Table 1:** Crucial differential metabolites identified in differently colored flaxseeds.

NO.	Compounds	Rt (min)	Q1 (Da)	Q3 (Da)	Main Fragments (Da)	Molecular Weight (Da)	Ionization Model	Formula	Class II
1	6-C-Glucosyl-5-deoxykaempferol	3.78	433.11	313.07	313.0712, 283.0622, 397.0834, 337.0734, 433.1108	432.1056468	[M + H]^+^	C_21_H_20_O_10_	Flavonols
2	Kaempferol-3-O-glucoside-7-O-rhamnoside *	3.95	595.17	287.06	287.0548, 449.1045, 85.0274, 129.0535	594.1585	[M + H]^+^	C_27_H_30_O_15_	Flavonols
3	Kaempferol-3-O-glucorhamnoside *	3.89	595.17	287.06	287.0548, 449.1045, 85.0274, 129.0535	594.1585	[M + H]^+^	C_27_H_30_O_15_	Flavonols
4	Kaempferol-3-O-neohesperidoside *	3.89	595.16	287.06	287.0548, 449.1045, 85.0274, 129.0535	594.1585	[M + H]^+^	C_27_H_30_O_15_	Flavonols
5	Kaempferol-3-O-robinobioside (Biorobin) *	3.89	595.1673	287.0551	287.0548, 449.1045, 85.0274, 129.0535	594.1585	[M + H]^+^	C_27_H_30_O_15_	Flavonols
6	Cyanidin 3-O-(6-O-p-coumaroyl)glucoside	3.89	595.14	287.06	287.0692	595.1446	[M]^+^	C_30_H_27_O_13_	Anthocyanidins
7	Vitexin	3.76	433.11	313.07	313.0712, 283.0622, 397.0834, 337.0734, 433.1108	432.1056	[M + H]^+^	C_21_H_20_O_10_	Flavones
8	Genistein 8-C-glucoside	3.77	433.11	283.06	313.0714, 283.0600, 397.0923, 337.0711, 433.1108	432.1056	[M + H]^+^	C_21_H_20_O_10_	Isoflavones
9	Luteolin-7-O-rutinoside *	3.94	595.17	287.06	287.0548, 449.1045, 85.0274, 129.0535	594.1585	[M + H]^+^	C_27_H_30_O_15_	Flavones
10	Scolymoside *	3.94	595.1658	287.0584	287.0548, 449.1045, 85.0274, 129.0535	594.1585	[M + H]^+^	C_27_H_30_O_15_	Flavones
11	2-(3,4-dihydroxyphenyl)-4h-chromene-3,5,7-triol-glucoside	3.31	451.12	243.06	243.0652, 271.0604, 215.0691, 149.0233, 289.0721	450.1162	[M + H]^+^	C_21_H_22_O_11_	Flavanols
12	2,6,7,4’-Tetrahydroxyisoflavanone	3.31	289.07	243.07	215.0701, 243.0657, 149.0231, 271.0595, 107.0486	288.0634	[M + H]^+^	C_15_H_12_O_6_	Isoflavones
13	Feruloyl syringic acid	3.45	375.11	137.1	137.0591	374.1002	[M + H]^+^	C_19_H_18_O_8_	Phenolic acids
14	Furo(2,3-f)-1,3-bewnzodioxole	3.31	271.06	149.02	271.0615, 215.0701, 149.0222, 243.0667, 253.0455	270.0528	[M + H]^+^	C_15_H_10_O_5_	Phenolic acids
15	Maesopsin 6-beta-D-glucopyranoside	3.31	451.12	289.07	243.0652, 271.0604, 215.0691, 149.0233, 289.0721	450.1162115	[M + H]^+^	C_21_H_22_O_11_	Aurones
16	(+)-Gallocatechin	2.37	307.08	139.04	139.0385, 163.0397, 181.0520, 223.0674	306.074	[M + H]^+^	C_15_H_14_O_7_	Flavanols
17	(−)-Epigallocatechin	2.74	307.08	139.04	139.0385, 163.0397, 181.0520, 223.0674	306.074	[M + H]^+^	C_15_H_14_O_7_	Flavanols
18	Phloretin 3’,5’-Di-C-glucoside	3.72	599.2	431.13	431.1336, 461.1441, 497.1475, 395.1156, 545.1679	598.1898	[M + H]^+^	C_27_H_34_O_15_	Flavones
19	1-(4-Hydroxybenzoyl)Glucose; 25545-07-7	2.69	299.08	93.03	137.0256, 93.0344, 136.0144, 59.0135, 71.0134	300.0845	[M − H]^−^	C_13_H_16_O_8_	Phenolic acids
20	1-O-p-Coumaroyl Octose	2.57	385.11	163.04	163.0398, 119.0503, 85.0297, 59.0140	386.1213	[M − H]^−^	C_17_H_22_O_10_	Phenolic acids
21	Salidroside	2.68	299.11	137.02	59.0139, 89.0249, 119.0483, 71.0139, 137.0631	300.1209	[M − H]^−^	C_14_H_20_O_7_	Phenolic acids
22	3-hydroxybenzoyl-β-D-glucose	2.68	299.08	137.02	137.0250, 93.0347, 71.0130, 299.0872	300.0845	[M − H]^−^	C_13_H_16_O_8_	Phenolic acids
23	6-O-Caffeoyl-D-glucose	2.84	341.09	179.03	179.0359, 135.0450, 59.0142, 341.0865	342.0951	[M − H]^−^	C_15_H_18_O_9_	Phenolic acids
24	Grevilloside F	2.52	341.09	135.04	179.0359, 135.0450, 59.0142, 341.0865	342.0951	[M − H]^−^	C_15_H_18_O_9_	Phenolic acids
25	2-hydroxynaringenin *	4.56	287.06	125.03	125.0242, 259.0608, 243.0653, 177.0552	288.0634	[M − H]^−^	C_15_H_12_O_6_	Flavanones
26	2-Hydroxy-2,3-dihydrogenistein *	4.56	287.06	125.02	125.0242, 259.0608, 243.0653, 177.0552	288.0634	[M − H]^−^	C_15_H_12_O_6_	Isoflavones
27	Drimiopsin C *	4.56	287.05	125.02	125.0242, 259.0608, 243.0653, 177.0552	288.0634	[M − H]^−^	C_15_H_12_O_6_	Other Flavonoids
28	Maesopsin *	4.56	287.05	125.03	125.0242, 259.0608, 243.0653, 177.0552	288.0634	[M − H]^−^	C_15_H_12_O_6_	Aurones
29	Kaempferol-3-O-rutinoside	3.91	593.15	285.04	593.1518, 285.0395, 284.0313	594.1585	[M − H]^−^	C_27_H_30_O_15_	Flavonols
30	2-O-Salicyl-6-O-Galloyl-D-Glucose	3.45	451.09	313.05	313.0543, 169.0125, 125.0229	452.0955	[M − H]^−^	C_20_H_20_O_12_	Phenolic acids
31	3,5-Di-Galloyl Sorbitol	1.78	485.09	315.07	315.0721, 169.0146, 152.0111, 125.0237, 423.0911	486.101	[M − H]^−^	C_20_H_22_O_14_	Phenolic acids

Note: Rt, retention time; Q1, precursor ion; Q3, product ion. Compounds marked with an asterisk (*) were putatively identified based on MS/MS fragmentation patterns and comparison with reference standards.

**Table 2:** ANOVA results for the eight candidate metabolites.

Compounds	Color Effect F-Value	Color Effect *p*-Value	FDR-Adjusted q-Value	Location Effect *p*-Value	Color × Location *p*-Value	Genotype (Color) *p*-Value	Direction
demethoxypinoresinol	18.27	<0.001	<0.001	0.056	0.213	0.047	↑ Yellow
phloretin 3′,5′-di-C-glucoside	19.48	<0.001	<0.001	0.234	0.067	0.042	↑ Brown
(−)-epigallocatechin	15.43	<0.001	0.002	0.201	0.076	0.031	↑ Brown
maesopsin 6-β-D-glucopyranoside	13.75	0.001	0.003	0.152	0.089	0.035	↑ Brown
(+)-gallocatechin	12.81	0.001	0.003	0.143	0.095	0.039	↑ Brown
3,5-di-galloyl sorbitol	11.96	0.002	0.004	0.078	0.112	0.028	↑ Brown
leucocyanidin	10.67	0.003	0.005	0.167	0.084	0.044	↑ Brown
gallate	9.84	0.004	0.006	0.189	0.102	0.038	↑ Brown

Note: F-values, *p*-values, and q-values were calculated from log_2_-transformed data. FDR adjustment was performed using the Benjamini–Hochberg method. Direction indicates whether the metabolite was more abundant in brown or yellow seeds.

## Data Availability

The original contributions presented in the study are included in the article/Supplementary Material; further inquiries can be directed to the corresponding authors.

## Supplementary Materials

The following supporting information can be downloaded at: https://www.mdpi.com/article/10.3390/foods15152690/s1, Figure S1: The TIC overlap (A, B) of QC specimens detected by MS and the XIC (C, D) of multi-peak maps for MRM metabolite detection; Figure S2: Correlation analysis of all the samples; Figure S3: PCA of the all cultivars; Figure S4: OPLS-DA permutation plot; Figure S5: Number of different types of differential metabolites for the comparative groups QC YJ2435 vs; Figure S6: Venn diagram of common and specific differential metabolites among 6 comparative groups; Table S1: The metabolites information of Flaxseed with Different Colors; Table S2: A list of differential metabolites between QC 2435 *VS.*QC 227, QC 2414 *VS.*QC 141, QC 2408 *VS.*QC 175, JT 2435 *VS.*JT 227, JT 2414 *VS.*JT 141, JT 2408 *VS.*JT 175; Table S3: A list of differential Polyphenol metabolites between QC 2435 *VS.*QC 227, QC 2414 *VS.*QC 141, QC 2408 *VS.*QC 175, JT 2435 *VS.*JT 227, JT 2414 *VS.*JT 141, JT 2408 *VS.*JT 175; Table S4: Differential metabolites significantly up‑regulated or down‑regulated in brown flaxseed compared with yellow flaxseed.
