# Supplementary material for: Widely Targeted Metabolomics Reveals Metabolic Differences Between Yellow and Brown Flaxseed Cultivars (Linum usitatissimum L.)
Source: Foods. 2026 Jul 30;15(15):2690. doi: 10.3390/foods15152690 (PMC13465061; doi:10.3390/foods15152690)
Supplement: Supplementary file 1 [file foods-15-02690-s001.zip › Figure_S1.pdf]

A

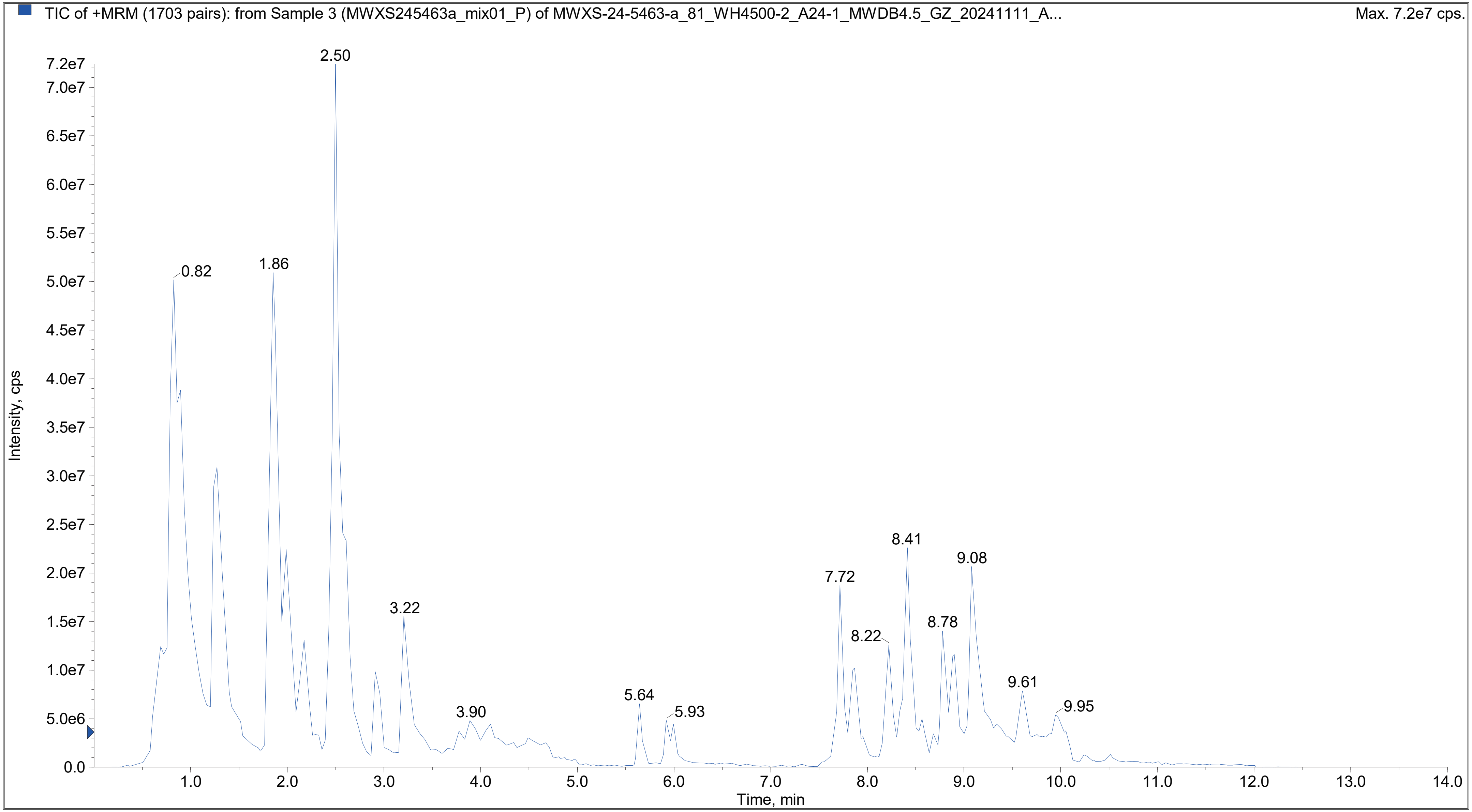
TIC of +MRM (1703 pairs): from Sample 3 (MWXS245463a_mix01_P) of MWXS-24-5463-a_81_WH4500-2_A24-1_MWDB4.5_GZ_20241111_A...
Max. 7.2e7 cps.
Intensity, cps
Time, min
7.2e7
7.0e7
6.5e7
6.0e7
5.5e7
5.0e7
4.5e7
4.0e7
3.5e7
3.0e7
2.5e7
2.0e7
1.5e7
1.0e7
5.0e6
0.0
1.0
2.0
3.0
4.0
5.0
6.0
7.0
8.0
9.0
10.0
11.0
12.0
13.0
14.0
2.50
0.82
1.86
3.22
3.90
5.64
5.93
7.72
8.22
8.41
8.78
9.08
9.61
9.95

B

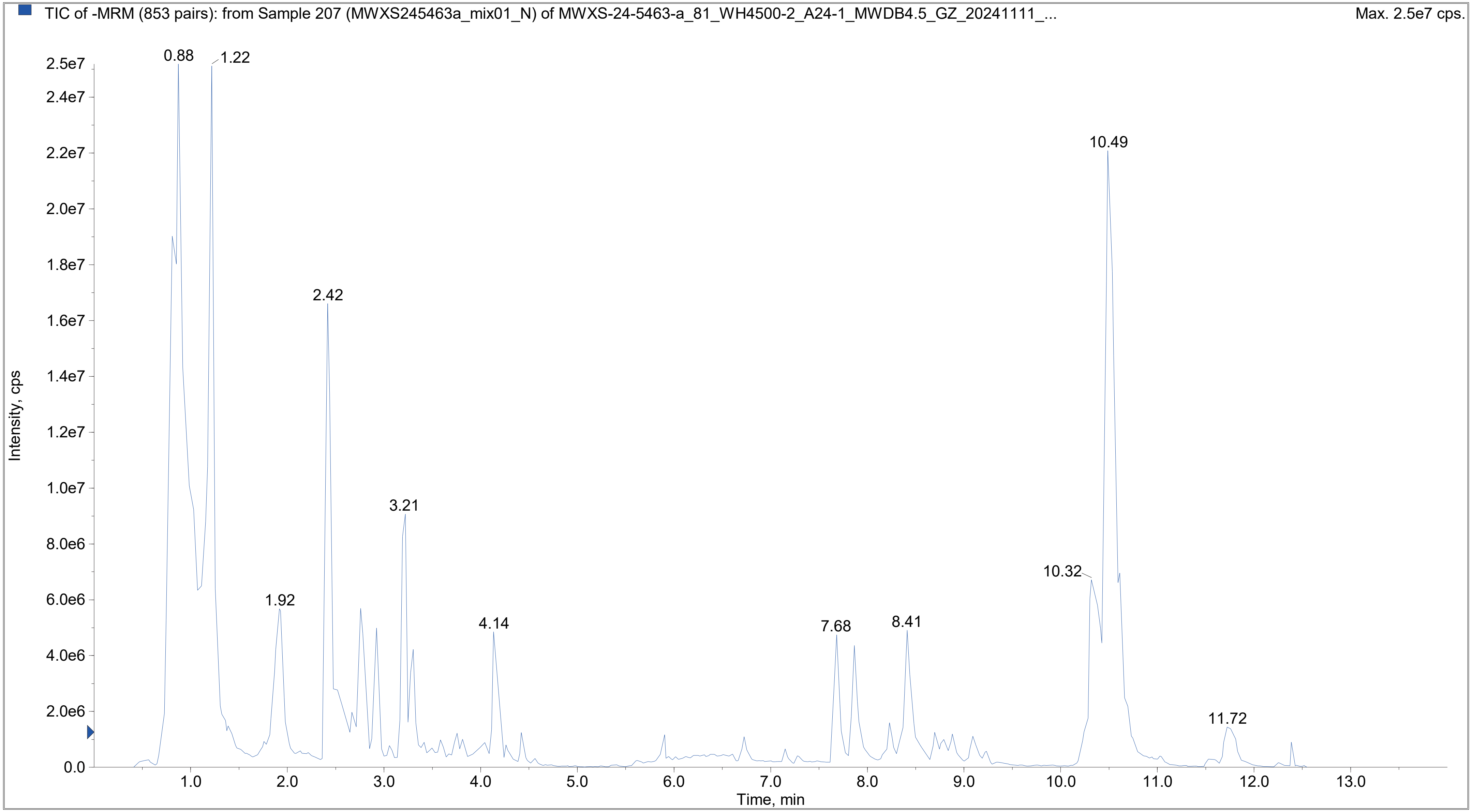

TIC of -MRM (853 pairs): from Sample 207 (MWXS245463a_mix01_N) of MWXS-24-5463-a_81_WH4500-2_A24-1_MWDB4.5_GZ_20241111_...
Max. 2.5e7 cps.
Intensity, cps
Time, min
0.88
1.22
1.92
2.42
3.21
4.14
7.68
8.41
10.32
10.49
11.72
2.5e7
2.4e7
2.2e7
2.0e7
1.8e7
1.6e7
1.4e7
1.2e7
1.0e7
8.0e6
6.0e6
4.0e6
2.0e6
0.0
1.0
2.0
3.0
4.0
5.0
6.0
7.0
8.0
9.0
10.0
11.0
12.0
13.0

C

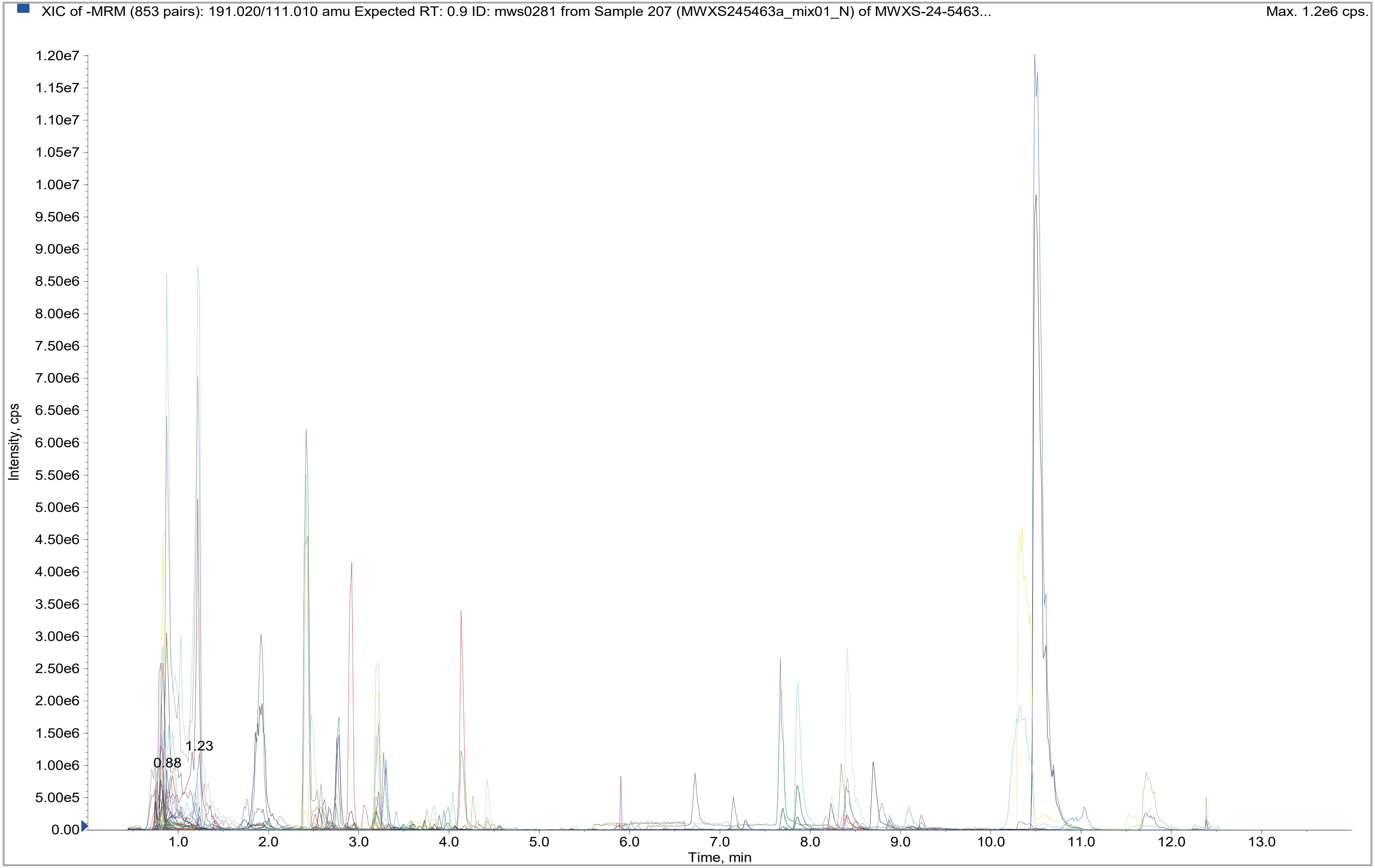

XIC of -MRM (853 pairs): 191.020/111.010 amu Expected RT: 0.9 ID: mws0281 from Sample 207 (MWXS245463a_mix01_N) of MWXS-24-5463...
Max. 1.2e6 cps.
Intensity, cps
1.20e7
1.15e7
1.10e7
1.05e7
1.00e7
9.50e6
9.00e6
8.50e6
8.00e6
7.50e6
7.00e6
6.50e6
6.00e6
5.50e6
5.00e6
4.50e6
4.00e6
3.50e6
3.00e6
2.50e6
2.00e6
1.50e6
1.00e6
5.00e5
0.00
1.23
0.88
1.0
2.0
3.0
4.0
5.0
6.0
7.0
8.0
9.0
10.0
11.0
12.0
13.0
Time, min

D

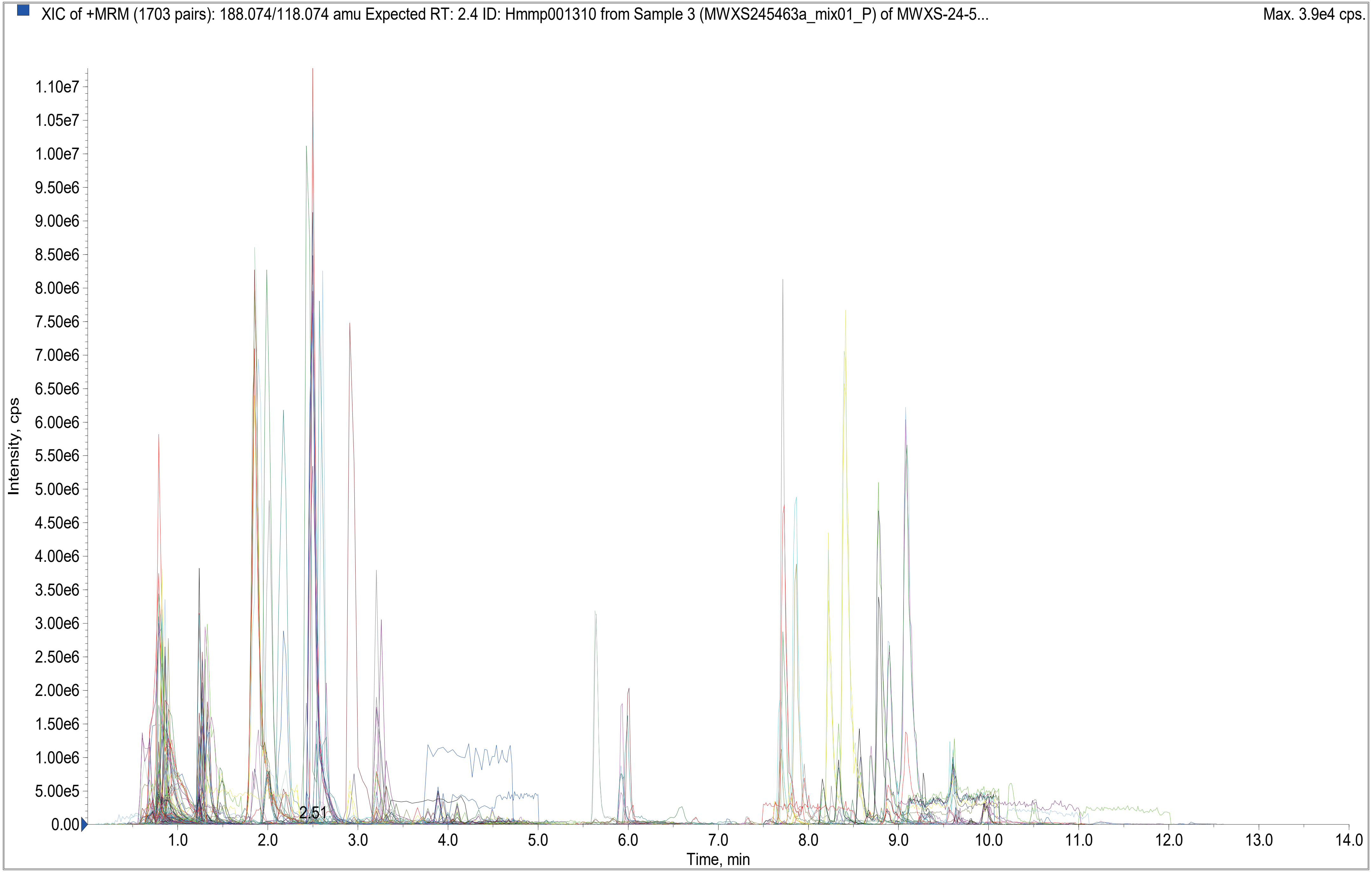
XIC of +MRM (1703 pairs): 188.074/118.074 amu Expected RT: 2.4 ID: Hmmp001310 from Sample 3 (MWXS245463a_mix01_P) of MWXS-24-5...
Max. 3.9e4 cps.
Intensity, cps
Time, min
2.51
1.10e7
1.05e7
1.00e7
9.50e6
9.00e6
8.50e6
8.00e6
7.50e6
7.00e6
6.50e6
6.00e6
5.50e6
5.00e6
4.50e6
4.00e6
3.50e6
3.00e6
2.50e6
2.00e6
1.50e6
1.00e6
5.00e5
0.00
1.0
2.0
3.0
4.0
5.0
6.0
7.0
8.0
9.0
10.0
11.0
12.0
13.0
14.0
