# Supplementary material for: Widely Targeted Metabolomics Reveals Metabolic Differences Between Yellow and Brown Flaxseed Cultivars (Linum usitatissimum L.)
Source: Foods. 2026 Jul 30;15(15):2690. doi: 10.3390/foods15152690 (PMC13465061; doi:10.3390/foods15152690)
Supplement: Supplementary file 1 [file foods-15-02690-s001.zip › Figure_S2.pdf]

A

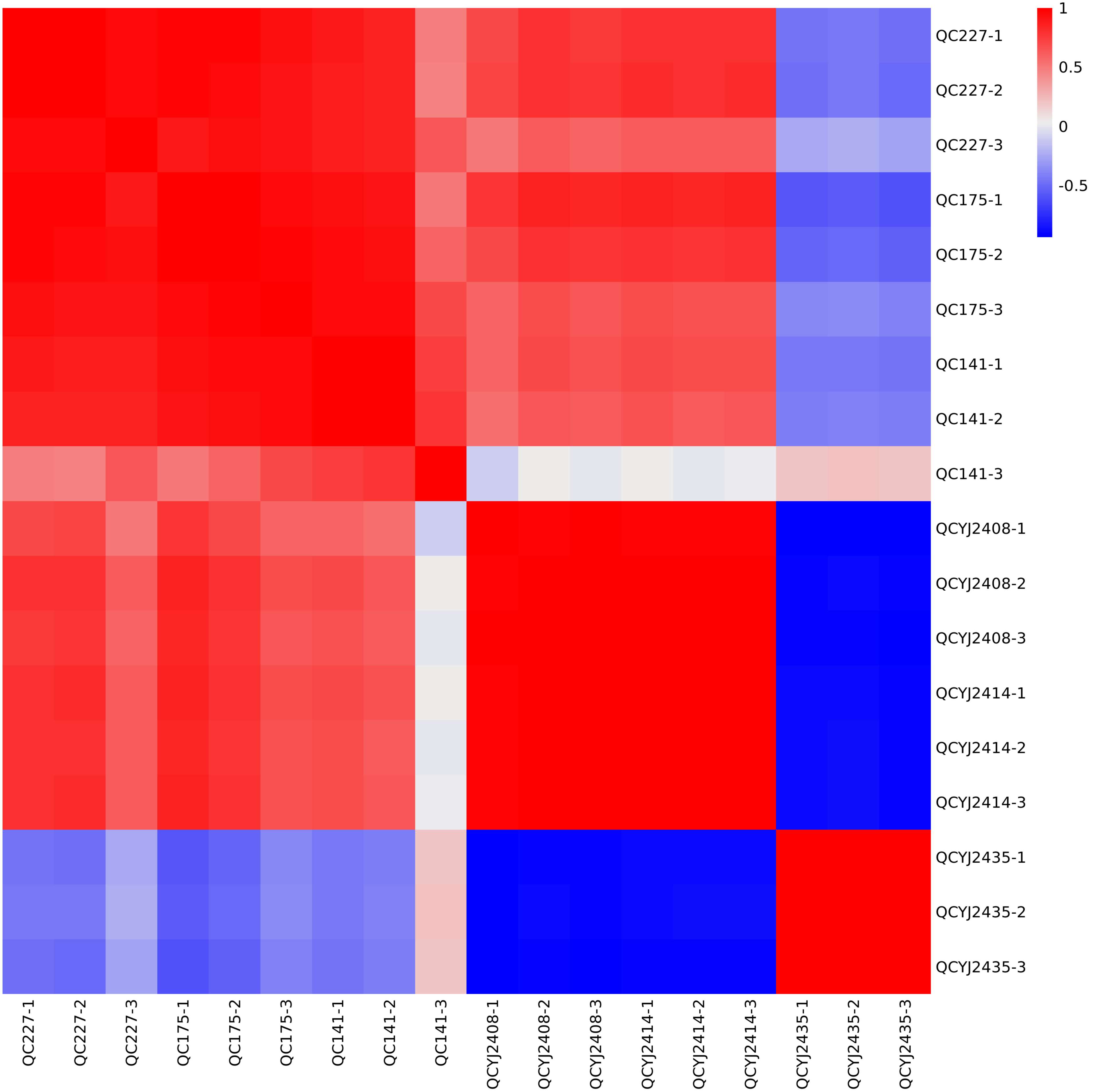
QC227-1
QC227-2
QC227-3
QC175-1
QC175-2
QC175-3
QC141-1
QC141-2
QC141-3
QCYJ2408-1
QCYJ2408-2
QCYJ2408-3
QCYJ2414-1
QCYJ2414-2
QCYJ2414-3
QCYJ2435-1
QCYJ2435-2
QCYJ2435-3
1
0.5
0
-0.5

B

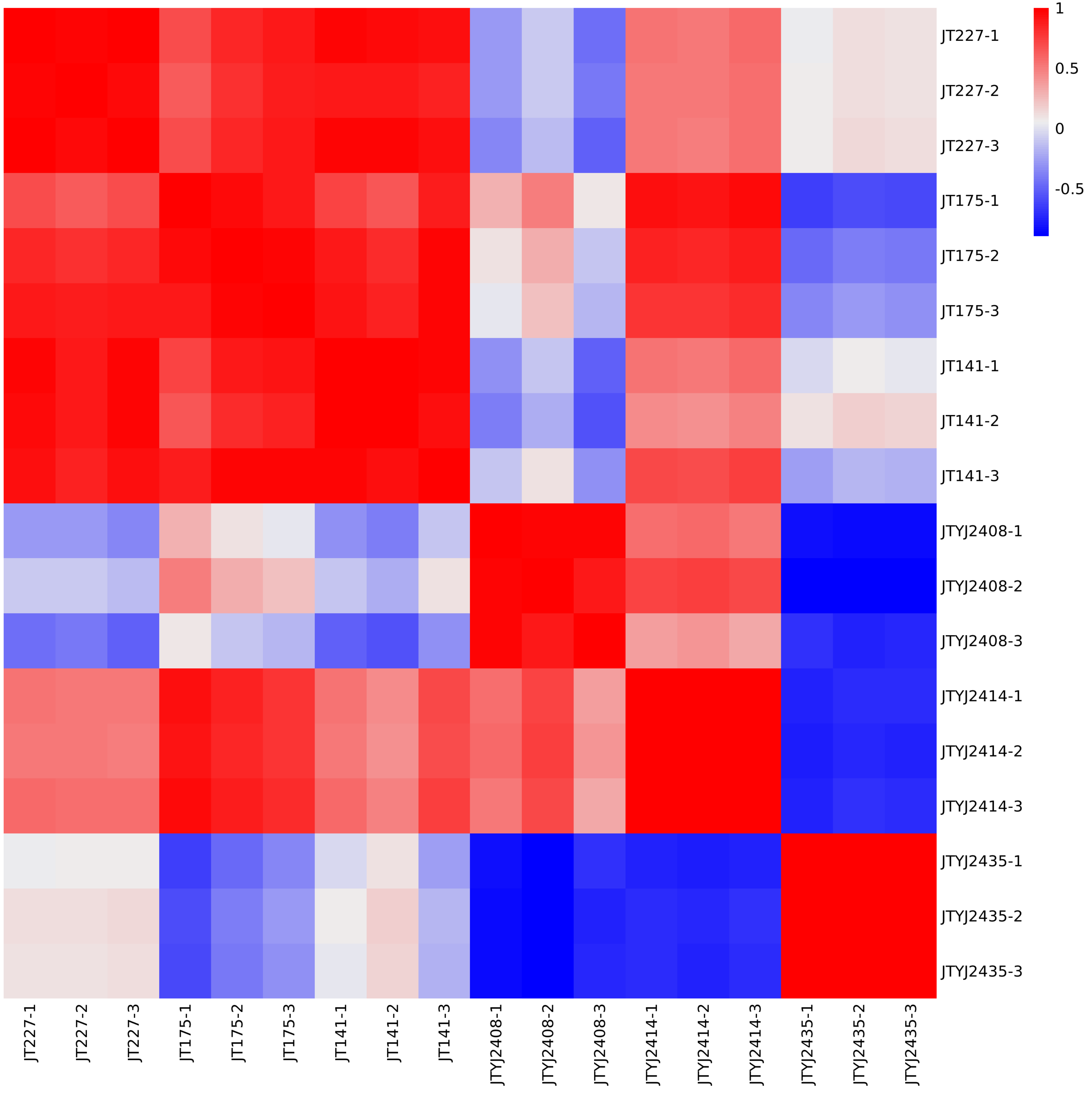
JT227-1
JT227-2
JT227-3
JT175-1
JT175-2
JT175-3
JT141-1
JT141-2
JT141-3
JTYJ2408-1
JTYJ2408-2
JTYJ2408-3
JTYJ2414-1
JTYJ2414-2
JTYJ2414-3
JTYJ2435-1
JTYJ2435-2
JTYJ2435-3
1
0.5
0
-0.5
